# Data Science and Plant Metabolomics

**DOI:** 10.3390/metabo13030454

**Published:** 2023-03-20

**Authors:** Anna Kisiel, Adrianna Krzemińska, Danuta Cembrowska-Lech, Tymoteusz Miller

**Affiliations:** 1 Institute of Marine and Environmental Sciences, University of Szczecin, Wąska 13, 71-415 Szczecin, Poland; 2 Polish Society of Bioinformatics and Data Science BIODATA, Popiełuszki 4c, 71-214 Szczecin, Poland; 3 Department of Physiology and Biochemistry, Institute of Biology, University of Szczecin, Felczaka 3c, 71-412 Szczecin, Poland

**Keywords:** plant metabolites, data science, machine learning

## Abstract

The study of plant metabolism is one of the most complex tasks, mainly due to the huge amount and structural diversity of metabolites, as well as the fact that they react to changes in the environment and ultimately influence each other. Metabolic profiling is most often carried out using tools that include mass spectrometry (MS), which is one of the most powerful analytical methods. All this means that even when analyzing a single sample, we can obtain thousands of data. Data science has the potential to revolutionize our understanding of plant metabolism. This review demonstrates that machine learning, network analysis, and statistical modeling are some techniques being used to analyze large quantities of complex data that provide insights into plant development, growth, and how they interact with their environment. These findings could be key to improving crop yields, developing new forms of plant biotechnology, and understanding the relationship between plants and microbes. It is also necessary to consider the constraints that come with data science such as quality and availability of data, model complexity, and the need for deep knowledge of the subject in order to achieve reliable outcomes.

## 1. Introduction

Plant metabolism encompasses the chemical reactions and processes happening in a plant’s cells to support life, growth, and reproduction. These encompass the transformation of energy and substances, such as water, carbon dioxide, and minerals, into parts of plants, such as starch and other storage products, cellulose, sugars, and various metabolites such as essential oils and allelochemicals [1]. This complex network contains photosynthesis, respiration, transpiration, and biosynthetic pathways which generate required metabolic intermediates, structural components, and various secondary metabolites [2]. The organic compounds produced in this way are usually divided by perspective function into primary metabolites, secondary metabolites (also called specialized metabolites or natural products), and plant hormones [3]. Primary metabolism products derived from glycolysis, the tricarboxylic acid (TCA) cycle, or the shikimate pathway often serve as precursors for the synthesis of the tens of thousands of secondary metabolites that have already been described [4]. Primary metabolites are highly conserved and directly required for plant growth and development [5] and secondary metabolites, including major groups such as phenolic compounds, terpenes, and nitrogen-containing compounds, are often lineage-specific and help plants interact with the biotic and abiotic environment [6].

Photosynthesis captures light energy and converts it into chemical energy in the form of adenosine triphosphate (ATP) and nicotinamide adenine dinucleotide phosphate, (NADPH), which are used to create sugars and starches. The process of respiration, by contrast, releases energy to power cellular processes through the conversion of organic compounds into CO_2_ and water while producing ATP [7].

In addition to basic metabolic pathways, plants also produce a wide range of secondary metabolites including allelochemicals, pigments, and essential oils. These compounds have vital functions in the defense system, signaling, communication, and environmental adaptation of the plant, and are made by complicated biosynthetic pathways which are regulated by multiple environmental and genetic elements [8].

However, it should be remembered that the boundary between primary and secondary metabolism is uncertain, e.g., because many primary metabolism intermediates play similar roles in secondary metabolism. Secondary metabolites were previously thought to only mediate plant-environment interactions, but recent genetic and chemical studies show they can also regulate plant growth and defense, blurring the boundaries between these groups. Combining the roles these compounds play in the plant provides a close link between primary and secondary metabolism, and the distinctions between these processes must be made with increasing caution. It may be necessary to revisit the existing functional division [9,10,11]. Viewing secondary metabolites as integrated components of metabolic networks shaped by environmental selection pressures can improve our understanding of plant metabolism and plant-environment interactions.

In addition, it is important to remember that plant metabolism is regulated by a complex system of enzymes and pathways that regulates plant metabolism, which is influenced by genetic and environmental conditions such as temperature, light, and nutrient availability [12]. Unraveling plant metabolism is critical for a variety of purposes, from establishing sustainable agronomic practices to increasing crop productivity and resilience to environmental extremes, to discovering new products and remedies derived from plants [2]. Overall, plant metabolism studies give us invaluable insight on how plants maintain their existence, how they adjust to environmental changes, and how these revelations can guide the creation of innovative approaches to boost productivity and sustainability with regards to vegetation-based products and medications [13,14].

Data science requires taking multiple steps, such as collecting and organizing data, exploring and visualizing it, engineering features, and constructing and validating models, to finally deploy and supervise their outcomes. For this purpose, data scientists rely on many tools and strategies such as machine learning algorithms, statistical models, data mining, and big data technologies, in addition to data visualization tools [15].

Data science has an extensive range of applications in many industries, such as finance, retail, healthcare, transportation, and the environment. It is employed to manage difficult issues, such as foreshadowing patron conduct, discovering frauds and irregularities, optimizing logistics networks, and rearing public health [16,17]. The field of data science is constantly progressing, necessitating an aptitude in mathematics and computation as well as a thorough awareness of the principles involved in data analysis and modeling [18]. All the while, new technologies, algorithms, and strategies are being devised for uses concerning all manners of raw information [19]. Domain knowledge holds immense importance in data science. This type of knowledge refers to expertise and familiarity with a particular field or industry, which is key when comprehending the intentions and aims of a data science endeavor. Furthermore, strong domain knowledge aids in interpreting and disseminating the outcomes of data science projects, as well as detecting any potential biases or constraints. Ultimately, domain knowledge is vital for enabling successful, impactful data science results [20].

The purpose of this study is to present the possibility of using different data science methods and techniques such as machine learning, network analysis, and statistical modeling to evaluate data from plant metabolism studies. As we know, plant metabolism is one of the most difficult areas of plant research due to the large number of metabolic pathways, their mutual interactions, and dependence not only on the genotype but also on the environment. Any tool that will facilitate the assessment of the huge amount of data obtained during the analysis of metabolism is worth the interest of scientists. The possibilities of using artificial intelligence in the study of plant metabolism, as well as understanding the interaction between plant metabolism and the environment, have not been sufficiently understood. The use of artificial intelligence should provide the ability to predict the impact of environmental factors on plant metabolism and optimize plant breeding programs. Based on the collected literature, plant metabolites were characterized in the context of their functions in the biology of plant systems and application possibilities, and an outline of methods used for plant metabolic profiling was provided. The following sections discuss the possibilities of using data science methods for mathematical modeling and explain the software tools available for simulation purposes. We then review the possibilities that recent discoveries in data science have opened up.

## 2. Plant Metabolites

### 2.1. Characteristics of Plant Metabolites and Its Applications

Primary metabolism is responsible for the production of appropriate compounds necessary for the survival of the plant, referred to as primary metabolites. As a result of reactions involving many enzymes, a wide range of molecules from the category of carbohydrates, amino acids, fatty acids, nucleic acids, and polymers derived from them (polysaccharides, proteins, lipids, etc.) are synthesized and used. Importantly, primary metabolites are identical in all living plant cells and are responsible for basic life functions such as respiration, growth, cell division, and reproduction [5]. Plant secondary metabolites, on the other hand, are formed from primary metabolites under the influence of various environmental stresses, such as light, temperature, and various metals, through several metabolic pathways. The formation of secondary metabolites is very specific to each family of plants, which from the same primary metabolites produce a large number of different secondary metabolites with different functions. They are mainly responsible for the interaction of the plant with the environment, hence their role in plant defense against biotic (viruses, bacteria, fungi, and insects) and abiotic (metals, temperature, light) stresses [21,22]. Among plant secondary metabolites, we can distinguish several basic sections based on chemical structure and functional groups. They include major groups such as terpenes, phenolic, polysaccharides, hydrocarbons, nitrogen-containing compounds, and sulfur-containing compounds [23] (Table 1).

Terpenes are a large, diverse group of plant secondary metabolites. Among them are such important substances as insect attractants, essential oils, growth inhibitors, and plant hormones such as gibberellic acid and abscisic acid. All terpenes have nascent five-carbon isoprene units [24]. They are classified according to the number of isoprene units in the molecule and the prefix in the name indicates the number of terpene units. Monoterpenes are allelochemicals present in the essential oil in plants. They can be found in such plant organs as fruits, leaves, bark, or stems of herbaceous plants [25]. These substances are therefore responsible for the appropriate smell of plants, which in this way attract pollinating insects and for deterring pests with it [26]. Some monoterpenes have antifungal and antibacterial properties. Many of them have an intense taste and smell [27]. They also have a special role in plant communication as infochemicals, enabling the propagation of defense signals between plants [28]. Camphor and menthol are used as anti-irritants, analgesics, and antipruritics; others have a coronary vasodilator effect [43]. Sesquiterpenes are compounds that are also attractive for pollinators and substances involved in plant communication using volatiles [29]. They exhibit antibacterial, antifungal, and antiprotozoal effects. They are also responsible for the healing properties of some plants, e.g., *Atracylodis macrocephala* [25,30]. In addition to therapeutic effects, sesquiterpenes may also have toxic and allergenic effects for humans and animals, which indicates their defensive function in the plant organism [30]. Diterpenes are very important compounds in herbal medicine. They exhibit a number of pharmacological properties: analgesic, antibacterial, diaphoretic, anti-inflammatory, and many others [25,44]. They are also involved in the basic elements of plant life, such as plant growth and development, and in the defense mechanism against pathogens [45]. Sesterterpens are a group of terpenes not yet fully explored, but researchers find these compounds in various parts of plants. It is assumed that they play an important role in the defense mechanism of plants against pathogens [32]. Some show cytotoxic activity in leukemic cells [46]. Triterpenes are compounds that can be divided into many subgroups, including saponins. Saponins are characterized by numerous therapeutic applications, but they are also surface-active substances that, in contact with water, form a foamy solution [33]. Simple triterpenes are components of surface waxes and specialized membranes and have the potential to act as signaling molecules [34]. Triterpene compounds are steroid precursors in both animals and plants and have anti-inflammatory and antirheumatic effects [47]. Polyterpenes are complex chemical compounds that occur in plants in the form of so-called resins and rubber. Rubber is a widely used material, an important element of the economy [35]. Polyterpene resins are used as a binder for various types of adhesives and in the production of paints [36].

Currently, it is believed that phenols are the largest group of secondary metabolites. They include both simple compounds with single aromatic rings and complex compounds such as tannins or lignins, which are polymers [24], and they share the presence of one or more phenol groups. These are compounds that perform many important functions in plants: among others, they are responsible for the color, taste, and smell of many plants. Phenolics are valued substances used in herbal medicine due to their anti-inflammatory effect [25]. Phenolic acids are ubiquitous in plants and the member gallic acid is well known for its astringent properties, although it has many other properties, including antiviral, antibacterial, antifungal, anti-inflammatory, and anticancer properties. Salicylates have anti-inflammatory properties [31] and phenol was used as the first antiseptic because it has antimicrobial activity [48]. Coumarins are chemical compounds that are very common in many plant species. Coumarin has been found in about 150 species belonging to more than 30 different families. They exhibit anti-inflammatory, antithrombotic, and anticancer properties, which makes them important substances in herbal medicine [25]. They are of great importance in plant nutrition. They are also responsible for the sweet smell and taste of plant organs, which is supposed to protect the plant from being eaten [49]. Furocoumarins act as a defense mechanism against mammals and insects. They also exhibit antifungal activity. These compounds can be found in roots, fruits, and leaves, often as components of essential oils. They are also responsible for the phytotoxic properties of plants such as *Datura* sp. or *Ruta* sp. [37]. Lignins are chemical compounds that are the main component of the cell walls of plant cells [38]. They are dimeric compounds found in many different species of plants. Many of the lignins have antimicrobial, antifungal, and cytotoxic effects such as wikstromal, matairesinol [25,50]. Resveratrol, on the other hand, has estrogen-like effects [51]. Flavonoids are a large group of compounds with considerable structural diversity. More than 2000 flavonoids are already known. The most common of these are anthocyanins, flavones, and flavonols. They are characterized by antioxidant, anti-inflammatory, antiallergic, antiviral, and anticancer effects. Therefore, they are of great importance in herbal medicine, and the research and use of these substances in dietetics and natural medicine are becoming more and more numerous. In plants, they are responsible for the colors of flowers and fruits [24,52,53]. Isoflavonoids are compounds that are very similar to flavonoids in their use as antioxidants, but these substances can also be classified as phytoestrogens that have the ability to bind to estrogen receptors. Phytoestrogenic activity excerpt as genistein and daidzein. This action can have a good effect on the body, but research on all aspects of their function is still in progress. In plants, they play a role in the defense mechanism, mainly against fungi [39,54]. Tannins are polyphenols that are very common in the plant kingdom. These compounds have been used for centuries to transform animal skins because they have the ability to precipitate protein [55]. Drugs containing tannins have an antidiarrheal effect and have been used as antidotes in poisoning with heavy metals and alkaloids, and as an antiseptic [25]. In plants, tannins can be found in the leaves, bark, or wood itself. They are closely related to plant defense mechanisms against herbivorous mammals and insects [40].

Nitrogen-containing compounds are substances having one or more nitrogen atoms, usually in a heterocyclic ring. These compounds are easily soluble in water, optically active and have a significant effect on animals [24]. Among these substances are well-known alkaloids such as caffeine, nicotine, cocaine, and morphine [41,56]. It is estimated that 50% of drugs and pharmaceuticals of plant origin are alkaloids [42]. In the plant they have a role in germination and of course protecting plants from predators. Alkaloids have a very pronounced effect on animals, including humans. Many of the alkaloids act on the nervous system, because highly addictive opioids are alkaloids. In addition to the negative effect, they are used in herbal medicine as analgesics [39]. Cyanogenic glucosides are very common substances in plants. Their function in plants depends on activation by β-glucosidases to release toxic volatile hydrogen cyanide (HCN) as well as ketones and aldehydes to ward off herbivores and pathogens [44]. Non-protein amino acids are structures used by plants in their interactions with bacteria, fungi, herbivores, and other plants. They are found in the plant flower nectar and rhizosphere. They are also classified as allelochemicals [57].

Sulfur-containing compounds are organic substances that, even in small amounts, promote, inhibit, or modify physiological or abiotic stress in plants [58]. They also exhibit antibacterial and antifungal activity, which indicates their role in the defense mechanism of plants against pathogens [59].

Polysaccharides are widely distributed in plant organs such as roots, leaves, shoots, and seeds with anticancer, antioxidant, hypoglycemic, antibacterial, and antiviral effects [48]. In plant organisms, they occur in the composition of cell walls as cellulose or pectin and as reserve substances in the form of starch or inulin [60].

Hydrocarbons are very simple chemical compounds, made of hydrogen and carbon. They exist as simple chains or rings and form the basic backbone of more complex molecules. The waxes that build the coating on leaves and fruits contain many unsaturated hydrocarbons that are insoluble in water. They prevent water from sticking to the surface of the leaves. Hydrocarbons are also found in olive oil. An important hydrocarbon in plant development is ethylene, which plays the role of a plant hormone. It causes the fruit to ripen, the leaves to drop, and the neighboring flowers to wilt [39].

Primary and secondary plant metabolites are of great economic importance. They have some common features, they can usually be extracted from plant material by steam distillation, organic, or aqueous solvents, and are low molecular weight (>2000 Da) compounds with the exception of i.a. starch, gums, pectins, and natural rubber biopolymers, condensed tannins [61,62]. Plant metabolites are used in many industries, including pharmacology and medicine [63,64,65], agriculture [66,67], food industries [68,69,70], and other industries, including textiles and cosmetics [71,72].

### 2.2. Methods of Testing Plant Metabolites

Metabolomics is the study of the composition of the pool of metabolites (metabolic profiling) present in every organism, including plants. Thanks to metabolomics, it is possible to understand of phenotypic expression of plants as well as study changes and the regulation of plant metabolism in order to understand their adaptive and defensive responses to environmental stress [73,74]. Metabolomics has been divided into two distinct approaches, untargeted (which is a less specific analysis of all measurable analytes in the sample) and targeted metabolomics (specific and sensitive analysis of defined and biochemically annotated metabolites). The quantity and complexity of metabolites and their characteristics make metabolomic studies extremely complex. It is necessary to use methodology and instruments to comprehensively identify and measure each metabolite [75,76].

The analysis of metabolites (primary or secondary) starts with sample preparation, which includes the extraction of metabolites by various methods. Among the extraction methods promising in metabolomic analysis are the methods of quenching, and mechanical and ultrasonic extraction, sometimes integrated [77,78]. The selection of solvents is also of key importance, of which chloroform, methanol, and water are most often mentioned [79,80,81]; it is necessary to extract and enrich the sample with interesting metabolites and to remove impurities such as proteins and salts that hinder the analysis. Extraction is performed using various methods, selecting the proportions of organic solvents, and also based on liquid-liquid extraction or solid phase extraction [82].

Many tools and techniques are used in metabolomics, and usually a combination of them. Mass spectrometry (MS) is one of most powerful and commonly used analytical methods in metabolomics, allowing a choice of sensitivity and resolution performance using either single (MS) or tandem (MS/MS) mass analyzers. A variety of MS-based techniques are now available for untargeted and targeted metabolic profiling using LC (liquid chromatography)-MS, GC (gas chromatography)-MS, CE (capillary electrophoresis)-MS, FTICR (Fourier transform ion cyclotron resonance)-MS, MALDI (matrix-assisted laser desorption/ionization), IMS (ion mobility spectrometry) and NMR (nuclear magnetic resonance). GC-MS achieves a higher separation of metabolites than LC-MS and avoids ion suppression by taking advantage of the gaseous phase and the nature of its MS ionization. However, otherwise for LC-MS, GC-MS requires chemical derivatization of the metabolic prior to the analysis. In turn ion mobility mass spectrometry is a gas-phase ion separation technique, which takes advantage of differences in the mobilities of ions by size, shape, charge, and the interaction with the inert gas under the influence of an electric field. Mass spectrometry with CE is a very good technique for separating polar ionic and charged substances that are separated based on their charge and size ratio in an aqueous medium. It enables significant efficiency in the analysis of metabolites in biological samples, especially for compounds with high polarity and water solubility. Furthermore, CE-MS is fast, uses small amounts of sample and solvents per analysis, and requires little time for sample preparation in comparison to GC-MS.

As mentioned previously, the main goal of metabolome profiling is the analysis of small molecules. Beyond mass spectrometry, nuclear magnetic resonance (NMR) is a good analytical platform used to analyze small molecules in metabolomics. NMR uses molecules of nuclear spin energy in the presence of a magnetic field. Moreover, NMR is very fast and non-destructive, requires little time for sample preparation, and provides highly repeatable results. Another spectroscopy method that allows for metabolite analysis is FTICR which, with MS, provides measures of metabolites in a couple min with minimal pre-detector separation and without ion dissociation. MALDI-MS is one of the mass spectrometric ionization techniques often chosen for the analysis of large biomolecules, especially proteins. However, it has been recognized as a potentially high-throughput method for the metabolome profiling [76,83,84,85,86].

Metabolomic studies can detect even thousands of possible specialized metabolites, which leads to the creation of large data sets. That is why it is a huge challenge to extract information about specialized metabolites from the huge amount of data generated during analyzes. This requires transforming the raw data into a numerical matrix and then applying statistical methods that will facilitate the comparison of all results across all samples. Several programs are available for the in silico analysis of the large amount of metabolite spectral data generated by various analytical instruments. They are often proposed by manufacturers of apparatus for metabolomic analyses. Many bioinformatics tools and spectral libraries are available for data pre-processing, including XCMS, METLIN, PRIMe, AMDIS, MetaboAnalyst, MetAlign, and others [76,85,87,88,89,90,91].

## 3. Does Data Science Can Help in Studying Plant Metabolites?

### 3.1. Data Science Methods

Data science methods are an essential part of addressing the challenges posed by dangerous plant metabolites and environmental issues. These methods involve using statistical, computational, and mathematical techniques to analyze large and complex datasets, enabling the identification of patterns, relationships, and trends that may not be immediately apparent from the raw data [92].

In order to elaborate on the subject of plant metabolites, it should be mentioned that a summary of statistical methods and dedicated software has already been published [76,93].

In a recent review article by Johnson et al. [93], various statistical methods and software tools commonly used in plant metabolomics research were summarized. The authors highlighted the importance of the preprocessing and normalization of raw data, as well as the selection of appropriate statistical tests for analyzing metabolite abundance changes between different samples or treatments.

Among the statistical methods and software packages discussed by the authors were multivariate analysis tools such as principal component analysis (PCA), partial least squares (PLS), and orthogonal projections to latent structures (OPLS). These techniques can help identify patterns in metabolite data that may be associated with specific biological factors, such as treatment conditions or genetic variation.

Piasecka [76] provided a comprehensive review of various analytical methods that can be used to detect changes in plant metabolomics in response to biotic and abiotic stresses. The author highlighted the importance of integrating different types of data, such as transcriptomic and proteomic data, with metabolite data to obtain a more comprehensive understanding of plant stress responses.

In terms of statistical methods and software, Piasecka [76] discussed various multivariate analysis techniques, such as PCA and hierarchical cluster analysis (HCA), which can be used to identify groups of metabolites that are strongly correlated and may be associated with specific stress conditions.

The methods used in data science include machine learning algorithms such as Random Forest, Gradient Boosting, Support Vector Machines, and Neural Networks [94]. Remote sensing technologies like hyperspectral imaging, thermal imaging, Light Detection and Ranging (LiDAR), and satellite imagery are used to analyze the environment [95,96] while predictive modeling techniques such as species distribution models [97], generalized linear models [98], time series models [99], and projection models [100] are used to forecast future trends.

Spatial analysis is another important method used in data science, which involves using tools such as geographic information system (GIS), geostatistics [101], Kriging [102], and spatial clustering [103] to analyze spatial data. Data visualization techniques like heat maps [104], choropleth maps [105], scatter plots [106], and time series [107] plots are used to present the results of data analysis in a more accessible and understandable way.

Data preprocessing and cleaning methods like data imputation [108], outlier detection [109], feature selection [110], and normalization [111] are used to ensure the accuracy and completeness of data. Molecular biology techniques like dPCR [112], qPCR [113], and DNA sequencing [114,115] are also used to analyze genetic and molecular data related to plant metabolites and their impact on the environment.

Climate modeling techniques, including Global Circulation Models [116], Earth System Models [117], and Regional Climate Models [118], are used to study the impact of climate change on the environment. Environmental impact assessments such as Life Cycle Assessment [119], Environmental Impact Assessment [120], and Ecological Footprint Analysis [121] are used to assess the impact of human activities on the environment and develop strategies to mitigate those impacts.

The methods are called “data science methods” because they are part of the broader field of data science, which involves analyzing data using statistics, computational methods, and mathematics. By analyzing and processing large, complex datasets, these methods can reveal patterns, relationships, and trends that are not readily apparent in the raw data [122].

These data science methods are used to analyze large and diverse datasets, such as satellite imagery [123], climate data [124], and molecular biology [125] data, in order to gain a better understanding of how harmful plant metabolites impact the environment and to develop targeted management strategies in the context of addressing dangerous plant metabolites and environmental issues [126]. By using these methods, scientists and researchers can turn large and complex datasets into actionable information that can inform decision-making and help to mitigate the impacts of dangerous plant metabolites on the environment.

Data science methods offer several ways to study plant metabolites and their impact on the environment. Predictive modeling can be used to predict the growth and development of different plant species and the production of various metabolites, such as allelochemicals and essential oils [127]. Metabolic pathway analysis can be performed using transcriptomics, proteomics, and metabolomics data to understand the biosynthesis and regulation of different plant metabolites. Gene expression analysis can also be used to study the regulation of metabolic pathways and identify the genes responsible for the production of specific metabolites [128,129]. Some plants contain cyanide or other poisons [130,131,132,133,134,135,136,137,138,139,140,141,142,143,144,145,146,147]. Machine learning-based classification algorithms can be used to classify cells of different plant species based on their metabolic profiles and predict the potential production of harmful metabolites, such as allelochemicals and cyanide. Data visualization tools can be used to visualize and compare the metabolic profiles of different plant species and identify trends and patterns in the data. Network analysis can be used to study the relationships between different metabolites and the enzymes and pathways involved in their biosynthesis and degradation [92,148,149].

Environmental monitoring data, such as satellite imagery and climate data, can be used to study the impact of environmental factors such as temperature and precipitation on plant metabolism and the production of specific metabolites. By applying these data science methods, researchers can gain a deeper understanding of plant metabolism and the impact of specific metabolites on the environment. This knowledge can inform the development of sustainable agriculture and land use practices, helping to mitigate the negative impact of plant metabolites on the environment [150].

The role of AI in the context of plant metabolism and the classification of plant metabolites is still in its early stages. However, there are some potential applications of AI in this field. For example, AI algorithms can be used to analyze large-scale metabolomic datasets, which can help to identify novel secondary metabolites and their functions. Additionally, machine learning algorithms can be used to classify and predict the functions of different metabolites based on their structural properties and other features. This can help to refine our understanding of the roles of different plant metabolites in plant growth, defense, and interactions with the environment.

Moreover, AI can also help in predicting and understanding the impact of environmental factors on plant metabolism. For example, AI can be used to model the impact of climate change on the production of secondary metabolites in plants, which can help us to predict and mitigate the potential effects of climate change on plant communities and ecosystems. Additionally, AI can also assist in designing and optimizing plant breeding programs for developing crop varieties with specific metabolite profiles that confer desirable traits, such as resistance to pests or diseases. Overall, AI has the potential to significantly contribute to the advancement of our understanding of plant metabolism and the classification of plant metabolites [11].

### 3.2. Data Science Techniques

In addition to studying plant metabolism, there are several other data science approaches that can be employed. These include techniques such as statistical analysis and machine learning algorithms, which can be applied to better understand how plants function.

1. Clustering analysis:

Clustering algorithms can be used to group plants based on their metabolic profiles and identify metabolic similarities and differences between species. For example, clustering analysis can be used to group plant species based on their production of specific allelochemicals, such as terpenes, and compare the metabolic profiles of invasive and native plant species [151].

2. Dimension reduction techniques:

Techniques such as principal component analysis (PCA) and multidimensional scaling (MDS) can be used to reduce the complexity of large datasets and identify the most important metabolic pathways and metabolites. For example, PCA can be used to identify the most important metabolic pathways responsible for the production of volatile organic compounds (VOCs) in plants [152].

3. Artificial Neural Networks (ANNs):

ANNs can be used to model complex relationships between environmental factors, such as temperature and light, and the production of specific metabolites, such as essential oils. For example, ANNs can be used to predict the production of essential oils in different plant species based on environmental variables, such as temperature, light, and precipitation [153].

4. Decision Tree analysis:

Decision Tree analysis can be used to identify the most important environmental and genetic factors that influence the production of specific metabolites. For example, Decision Tree analysis can be used to identify plant species, which has implications for identifying the most important environmental and genetic factors that influence the production of allelochemicals in different plant species [154].

5. Bayesian networks:

Bayesian networks can be used to model the relationships between different metabolites and the pathways involved in their biosynthesis and degradation. For example, Bayesian networks can be used to model the relationships between different metabolites in the biosynthesis of secondary metabolites, such as flavonoids, and the enzymes and pathways involved in their production [149]. 

Examples of data science in plant metabolomic scientific field are presented in Table 2. 

## 4. The Advantages and Applications of Data Science in Plant Metabolism Studies

Data science has many potential benefits in the study of plant metabolism. One of the primary advantages is the ability to derive data-driven insights from complex biochemical processes and interactions that would be difficult to discern without the use of advanced analytical tools. Predictive modeling is another advantage of data science, enabling the development of models that can simulate and predict the behavior of plant metabolism under different conditions. These models can help identify new targets for intervention, optimize growth conditions, and improve crop yields [148,152].

Data science also enables the development of personalized solutions that take into account the unique biology and environment of each plant, leading to more targeted and effective interventions. Additionally, data science can integrate data from multiple sources and scales, providing a comprehensive understanding of plant metabolism, from molecular and cellular data to whole-plant and ecosystem data [162,163].

Sustainable solutions can also be developed using data science, including efficient irrigation and fertilizer application, which can help minimize costs and optimize resource utilization while also improving crop yields. Finally, data science can support decision-making by generating evidence-based recommendations for interventions and management strategies [164].

The benefits of data science in plant metabolism studies demonstrate its potential to revolutionize our understanding of plant biology, inform the development of innovative solutions, and improve decision-making processes. By utilizing data science, we can develop sustainable practices, optimize resource utilization, and improve crop yields, leading to a more efficient and environmentally responsible agricultural industry [165,166].

## 5. Challenges and Considerations for Data Science in Plant Metabolic Studies

The benefits of data science methods offer numerous benefits in plant metabolic studies, but it is important to consider the challenges and possible problems that come with their use. One of the primary challenges is the quality and availability of data. The difficulty in collecting and processing data at different scales and levels of detail, or the complexity of the underlying biology, can limit data quality and availability, making it challenging to generate accurate models [167].

Model complexity is another consideration, particularly when modeling complex biological systems like plant metabolism. Complex models can be difficult to interpret and communicate to stakeholders [168]. Overfitting is also a potential issue, particularly when data is limited, or the model is too complex to capture the underlying biological processes [169].

A lack of domain knowledge can lead to inaccurate or inappropriate models or result in misinterpreted results. Data science requires a combination of technical and domain knowledge, and a lack of the latter can significantly impact the accuracy of models and analyses [170].

Computational resources can also be a challenge, particularly with large and complex data sets. Data science methods can be computationally intensive, requiring significant resources to process and analyze data. In plant metabolic studies, where data sets can be especially large and complex, this can be particularly challenging [171].

While data science methods have numerous benefits in plant metabolic studies, it is essential to consider the challenges and address them appropriately. By addressing these considerations, it is possible to improve the accuracy, interpretability, and impact of data science methods, leading to more effective and informed plant metabolic research.

## 6. Conclusions

In this review, we have explored the potential of integrating data science techniques with plant metabolism studies to enhance our understanding of plant biology and its interactions with the environment. Our main findings indicate that machine learning algorithms, network analysis, and statistical modeling can contribute to new insights into plant growth, development, and response to environmental changes. Moreover, the application of these methods can help address the impact of environmental factors on metabolite production and the environmental consequences of plant metabolites.

By discussing various data science methods, including clustering analysis, dimension reduction, artificial neural networks, decision tree analysis, and Bayesian networks, this review expands the current knowledge in the field by demonstrating the versatility of data science techniques in plant metabolism research. Furthermore, we have shown how AI can be used to identify novel secondary metabolites, predict and understand the impact of environmental factors on plant metabolism, and optimize plant breeding programs.

As we look towards the future, several prospects emerge for further research and development. The integration of novel data sources, such as remote sensing and high-throughput phenotyping, can provide additional layers of information to enhance plant metabolism studies. The continued development of advanced machine learning techniques, such as deep learning and reinforcement learning, can lead to the more accurate and efficient modeling of complex biological systems. Additionally, interdisciplinary collaboration between plant scientists, data scientists, and other stakeholders will be crucial for addressing the challenges of data integration, model interpretability, and the ethical use of AI in plant research.

While some limitations and challenges persist, our review highlights the exciting potential of combining data science and plant metabolism studies. By fostering interdisciplinary collaboration, we can further advance plant biotechnology, sustainable agriculture, and our understanding of the complex interactions between plants and microbes. This synthesis of knowledge ultimately opens up new avenues for research with a promising future in addressing global food security and environmental sustainability.

## Figures and Tables

**Table 1:** Secondary metabolites in plants.

Classification	Types	Examples	Function in Plant	Ref.
Terpenes	Monoterpenes	geraniol, limonene, carvone, linalool, linalyl acetate, camphora	attract pollinating insects, deterring pests, antifungal and antibacterial activity, plant communication	[19,20,21,22]
	Sesquiterpenes	humulene, farnesol, bisabolol, caryophyllene, helenalin	plant communication, antibacterial, antifungal and antiprotozoal activity, healing	[19,23,24]
	Diterpenes	cafestol, placytaksol, ginogolide, taxane, aconane	plant growth and development, defense from pathogens	[19,25]
	Sesterterpenes	geranylfarneso, ophiobolin A, genepolide, gentianelloid A	defense fromst pathogens	[26]
	Triterpenes	squalene, cucurbitacin, oleane, ursolic acid, chamaecydin	signaling molecules	[27,28]
	Polyterpenes	gutta-percha	defense from herbivores	[29,30]
Phenolic	Simple phenolic	phenol, gallic acid, salicylic, ferulic and caffeic acids, hydroquinone	antimicrobial	[31]
	Coumarin	hydroxycoumarins, umbelliferone, esculetin, scopoletin	defense from insects, antifungical activity, role in plant nutrition, response in Fe stress	[19,30]
	Furanocoumarins	psoralin, angelicin, bergapten, methoxsalen	defense mechanism against mammals and insects, antifungal activity	[32]
	Lignin	resveratrol, wikstromal, matairesinol, dibenzyl, butyrolactol	antimicrobial, antifungal and cytotoxic effects	[19,33]
	Flavonoids	quercetins, luteolin, apigenin, peonidin, delphinidin	UV protection, pigmentation, antimicrobial defense, antioxidant activity, signal transduction, allelopathy, defense against herbivory, regulating gene expression, mediating symbiotic interactions	[18]
	Isoflavonoids	genistein, daidzein, ekwol, doumestrol, pueraryne	defense mechanism, mainly against fungi	[34]
	Tannins	tannic acid, geranilin, tellmagrandin 1 and 2	plant defense mechanisms against herbivorous, mammals, and insects	[19,35]
N containing compounds	Alkaloids	cocaine, nicotine, morphine, strychnine, codeine	role in germination, plants defense from predators	[18,34,36]
	Cyanogenic glucosides	linamarin, dhurrin, amygdalin, frunasin	ward off herbivores and pathogens	[37]
	Non-protein amino acids	L-Mimosine, L-Canavanine, 5-Hydroxy-L-tryptophan, L-3,4-Dihydroxyphenylolanine	interactions with bacteria, fungi, herbivores and other plants	[38]
S containing compounds		glutathione, glucosinolates, phytoalexins, thionins, defensins, allinim	physiological od abiotic stress, antibacterial and antifungal activity	[39,40]
Polysaccharides		pectin, celulose, inuline, alginian, starch	antibacterial and antifungal activity, plant cell walls components and starch components	[41,42]
Hydrocarbons		ethylene, march gas, methane	plant hormone—plant development	[34]

**Table 2:** Examples of data science in plant metabolomic scientific field.

Used Methods	Method	Study
Evaluating the physiological and biochemical responses of melon plants to NaCl salinity stress using supervised and unsupervised statistical analysis	OPLS-DA	Use of OPS-DA and PCA to predict melon plant response to salinity [152].
Ionomic and metabolomic analyses reveal the resistance response mechanism to saline-alkali stress in *Malus halliana* seedlings	OPLS-DA	Use of OPLS-DA to determine the nature of metabolic changes in leaves of apple seedlings [128].
Predicting metabolic pathways of plant enzymes without using sequence similarity: models from machine learning	mApLe	Using mApLe to predict metabolic pathways of plant enzymes instead of Enzyme Commission (EC) numbers [155].
Salinity source alters mineral composition and metabolism of *Cichorium spinosum*	OPLS-DA	Use OPLS-DA for the visualization of the fluctuations in the plant’s metabolome in response to the various treatments [156].
Physiological and metabolic responses triggered by omeprazole improve tomato plant tolerance to NaCl stress	OPLS-DA	Use OPLS-DA to separate the variability between the groups of samples [157].
Metabolic responses to potassium availability and waterlogging reshape respiration and carbon use efficiency in oil palm	OPLS	Use of OPLS to determine the significance of metabolome and proteome data components in the organs of the studied plants [158].
Comprehensive meta-analysis and machine learning approaches identified the role of novel drought specific genes in *Oryza sativa*	* SVM, kNN, NB, DT, RF	These machine learning techniques were used to identify the distinguishing features between test samples and controls based on accuracy [129,149].
Evaluating the physiological and biochemical responses of melon plants to NaCl salinity stress using supervised and unsupervised statistical analysis	PCA	Use of PCA to predict melon plant response to salinity [152].
	HCA	Use HCA to make a heat map to predict melon plant response of salinity [152].
Ionomic and metabolomic analyses reveal the resistance response mechanism to saline-alkali stress in *Malus halliana* seedlings	PCA	Use of PCA to predict variability in two groups of metabolites in leaf samples [128].
Principal component analysis of hormone profiling data suggests an important role for cytokinins in regulating leaf growth and senescence of salinized tomato	PCA	Using PCA as a mathematical tool to evaluate the relationship between physiological and hormonal variables in tomato research [159].
Salinity source alters mineral composition and metabolism of *Cichorium spinosum*	HCA	Use HCA to support OPLS-DA in the visualization of the fluctuations in the plant’s metabolome in response to the various treatments [157].
Physiological and metabolic responses triggered by omeprazole improve tomato plant tolerance to NaCl stress	PCA	Use PCA for obtaining a broad overview of morphological and physiological changes in tomato plants in response to the use of omeprazole in salted and unsalted conditions [157].
Changes in carbohydrates triggered by low temperature waterlogging modify photosynthetic acclimation to cold in *Festuca pratensis*	PCA	Use of PCA to determine the variability between parameters and to highlight the most important ones from the research perspective [160].
Zinc stress affects ionome and metabolome in tea plants	PCA	Using PCA for tissue ionome variation [161].
	HCA	Using HCA to visualize correlations between elements and metabolites in tea leaves [161].

* SVM—support vector machines, kNN—*k*-nearest neighbors algorithm, NB—naive Bayes classifiers, DT—decision tree, RF—random forest.
